# SARS-CoV-2 infection and the brain: direct evidence for brain changes in milder cases

**DOI:** 10.1038/s41392-022-01072-1

**Published:** 2022-07-11

**Authors:** Nico Sollmann, Ambros J. Beer, Frank Kirchhoff

**Affiliations:** 1 Department of Diagnostic and Interventional Radiology, Ulm University Medical Center, Ulm, Germany; 2 Department of Nuclear Medicine, Ulm University Medical Center, Ulm, Germany; 3 Institute of Molecular Virology, Ulm University Medical Center, Ulm, Germany

**Keywords:** Infectious diseases, Inflammation

**Dear Editor,**

A recent study published in *Nature* by Douaud and colleagues^1^ shows that SARS-CoV-2 infection is associated with longitudinal effects, particularly on brain structures linked to the olfactory cortex, modestly accelerated reduction in global brain volume, and enhanced cognitive decline. Thus, even mild COVID-19 can be associated with long-lasting deleterious effects on brain structure and function.

Loss of smell and taste are amongst the earliest and most common effects of SARS-CoV-2 infection. In addition, headaches, memory problems, confusion, or loss of speech and motility occur in some individuals.^2^ While important progress has been made in understanding SARS-CoV-2-associated neurological manifestations, the underlying mechanisms are under debate and most knowledge stems from analyses of hospitalized patients with severe COVID-19.^2^ Most infected individuals, however, develop mild to moderate disease and recover without hospitalization. Whether or not mild COVID-19 is associated with long-term neurological manifestations and structural changes indicative of brain damage remained largely unknown.

**Fig. 1:**
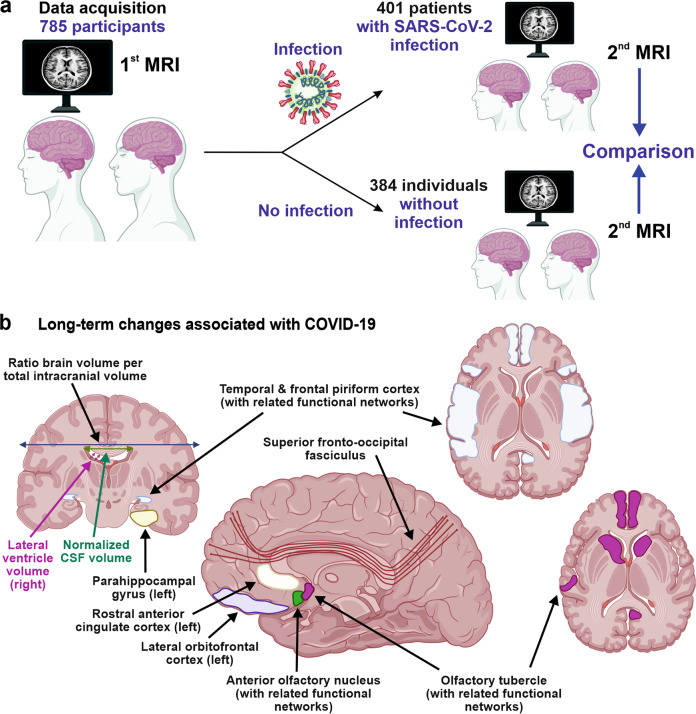
Identification of long-term changes associated with COVID-19. **a** Data acquisition: Two cerebral MRI acquisitions were performed with a mean inter-scan interval of 3.2 years in patients with and without intermittent SARS-CoV-2 infection (*n* = 384 patients without SARS-CoV-2 infection and *n* = 401 patients with SARS-CoV-2 infection). The first MRI acquisitions were then compared to the second MRI acquisitions using multi-parametric analyses. **b** Changes associated with COVID-19: Longitudinal changes between control subjects and patients with SARS-CoV-2 infection comparing the first and second MRI acquisitions. Based on a hypothesis-driven analysis (*n* = 297 olfactory-related cerebral imaging-derived phenotypes [IDPs]) as well as an exploratory analysis of changes beyond the olfactory system (*n* = 2047 IDPs), the ten respective main findings indicative of impaired brain structure and function are illustrated: ratio brain volume/estimated total intracranial volume, brain volume without ventricles & supratentorial volume without ventricles (dark blue arrow); normalized cerebrospinal fluid (CSF) volume (green arrow); lateral ventricle volume (right; purple arrows); parahippocampal gyrus (left; dark yellow); temporal and frontal piriform cortex (light blue, with related functional networks); olfactory tubercle (pink, with related functional networks); anterior olfactory nucleus (green; with related functional networks, not highlighted in the figure); lateral orbitofrontal cortex (left; purple); rostral anterior cingulate cortex (left; light yellow); superior fronto-occipital fasciculus (brown, illustrated as schematic streamlines representative of fibers); and crus II of the cerebellum (not shown). Adapted from “Neuroscience” and “COVID-19” by BioRender.com (2022). Retrieved from https://app.biorender.com/biorender-templates

Douaud and co-workers examined 785 participants of the UK Biobank (www.ukbiobank.ac.uk) who underwent magnetic resonance imaging (MRI) twice with an average inter-scan interval of 3.2 years, and 401 individuals testing positive for SARS-CoV-2 infection between MRI acquisitions (Fig. 1a). Strengths of the study are the large number of samples, the availability of scans obtained before and after infection, and the multi-parametric quantitative analyses of serial MRI acquisitions.^1^ These comprehensive and automated analyses with a non-infected control group allowed the authors to dissect consistent brain changes caused by SARS-CoV-2 infection from pre-existing conditions. Altogether, the MRI scan processing pipeline used extracted more than 2,000 features, named imaging-derived phenotypes (IDPs), from each participant’s imaging data. Initially, the authors focused on IDPs involved in the olfactory system. In agreement with the frequent impairment of smell and taste in COVID-19, they found greater atrophy and indicators of increased tissue damage in the anterior cingulate cortex, orbitofrontal cortex and insula, as well as in the ventral striatum, amygdala, hippocampus and para-hippocampal gyrus, which are connected to the primary olfactory cortex (Fig. 1b). Taking advantage of computational models allowing to differentiate changes related to SARS-CoV-2 infection from physiological age-related brain changes (e.g. decreases of brain volume with aging),^3^ they also explored IDPs covering the entire brain. Although most individuals experienced only mild symptoms of COVID-19, the authors detected an accelerated reduction in whole-brain volume and more pronounced cognitive declines associated with increased atrophy of a cognitive lobule of the cerebellum (crus II) in individuals with SARS-CoV-2 infection compared to the control group. These differences remained significant when 15 people who required hospitalization were excluded. Most brain changes for IDPs were moderate (average differences between the two groups of 0.2–2.0%, largest for volume of parahippocampal gyrus and entorhinal cortex) and accelerated brain volume loss was “only” observed in 56–62% of infected participants. Nonetheless, these results strongly suggest that even clinically mild COVID-19 might induce long-term structural alterations of the brain and cognitive impairment.

The study provides unique insights into COVID-19-associated changes in brain structure. The authors took great care in appropriately matching the case and control groups, making it unlikely that observed differences are due to confounding factors, although this possibility can never be entirely excluded. The mechanisms underlying these infection-associated changes, however, remain to be clarified. Viral neurotropism and direct infection of cells of the olfactory system, neuroinflammation and lack of sensory input have been suggested as reasons for the degenerative events in olfactory-related brain structures and neurological complications.^4^ These mechanisms are not mutually exclusive and may synergize in causing neurodegenerative disorders as consequence of COVID-19.

The study participants became infected between March 2020 and April 2021, before the emergence of the Omicron variant of concern (VOC) that currently dominates the COVID-19 pandemic. During that time period, the Alpha and Beta VOCs dominated in the UK and all results were obtained from individuals between 51 and 81 years of age. It will be of great interest to clarify whether Omicron, that seems to be less pathogenic than other SARS-CoV-2 variants, also causes long-term brain damage. The vaccination status of the participants was not available in the study^1^ and it will be important to clarify whether long-term changes in brain structure also occur in vaccinated and/or younger individuals. Other important questions are whether these structural changes are reversible or permanent and may even enhance the frequency for neurodegenerative diseases that are usually age-related, such as Alzheimer’s, Parkinson’s or Huntington’s disease. Previous findings suggest that cognitive disorders improve over time after severe COVID-19;^5^ yet it remains to be determined whether the described brain changes will translate into symptoms later in life such as dementia. Douaud and colleagues report that none of top 10 IDPs correlated significantly with the time interval between SARS-CoV-2 infection and the 2^nd^ MRI acquisition, suggesting that the observed abnormalities might be very long-lasting.

Currently, many restrictions and protective measures are relaxed because Omicron is highly transmissible but usually causes mild to moderate acute disease. This raises hope that SARS-CoV-2 may evolve towards reduced pathogenicity and become similar to circulating coronaviruses causing mild respiratory infections. More work needs to be done to clarify whether the current Omicron and future variants of SARS-CoV-2 may also cause lasting brain abnormalities and whether these can be prevented by vaccination or therapy. However, the finding by Douaud and colleagues^1^ that SARS-CoV-2 causes structural changes in the brain that may be permanent and could relate to neurological decline is of concern and illustrates that the pathogenesis of this virus is markedly different from that of circulating human coronaviruses. Further studies, to elucidate the mechanisms underlying COVID-19-associated neurological abnormalities and how to prevent or reverse them are urgently needed.
